# Supplementary material for: Perspectives of women, healthcare providers and health managers on Group Antenatal Care implementation in Geita, Tanzania: A qualitative study
Source: PLoS One. 2026 Apr 15;21(4):e0345539. doi: 10.1371/journal.pone.0345539 (PMC13082642; doi:10.1371/journal.pone.0345539)
Supplement: S1 Table — (DOCX) [file pone.0345539.s001.docx]

**Consolidated criteria for reporting qualitative research (COREQ)**

**Domain 1: Research team and reflexivity**

| **Item** | **Response** |
| --- | --- |
| **1. Interviewer/facilitator** | The interviews and focus group discussions were conducted by trained research assistants affiliated with the project, who were fluent in Kiswahili and English. |
| **2. Credentials** | The research team included professionals with advanced degrees such as MPH and MSc, and clinical and public health backgrounds in midwifery, nursing, and medicine. Research assistants were trained graduates in health-related fields. |
| **3. Occupation** | The researchers were employed as staff of the Prime Health Initiative Tanzania (PHIT) and Ministry of Health. Research assistants worked in health research and data collection roles. |
| **4. Gender** | Both male and female researchers were involved. |
| **5. Experience and training** | Data collectors were trained in qualitative interviewing techniques and ethical research conduct. The lead investigators had prior experience in qualitative health research. |
| **6. Relationship established** | Some of the facilitators were known to healthcare providers through previous training and collaboration in implementing the G-ANC, though no prior relationship existed with participating women. |
| **7. Participant knowledge of the interviewer** | Participants were informed about the purpose of the study, the voluntary nature of their participation, and the role of the researchers as evaluators of the G-ANC model. |
| **8. Interviewer characteristics** | The interviewers were perceived as neutral facilitators. Although interested in understanding G-ANC implementation, they used semi-structured guides with open-ended questions and actively encouraged participants to share both positive and negative experiences. |

**Domain 2: Study design**

| **Item** | **Response** |
| --- | --- |
| **9. Methodological orientation and theory** | Thematic analysis guided by Bowen et al.'s (2009) feasibility framework was used to analyze the data. |
| **10. Sampling** | A combination of purposive and snowball sampling was used. Facilities were selected based on geographic diversity, and women who attended G-ANC were recruited via snowballing. |
| **11. Method of approach** | Participants were approached face-to-face in their communities or during clinic visits. |
| **12. Sample size** | A total of 6 FGDs were conducted with 8–12 participants each, and 15 KIIs were held with healthcare providers and facility managers. |
| **13. Non-participation** | The manuscript does not specify instances of non-participation or dropouts. |
| **14. Setting of data collection** | Data were collected in private areas within health facilities to ensure comfort and confidentiality. |
| **15. Presence of non-participants** | No; only participants and the research team (moderator and note-taker) were present during data collection. |
| **16. Description of sample** | The sample included recently delivered women and pregnant women attending G-ANC, as well as ANC providers, facility in-charges, and health managers from rural, peri-urban, and urban settings. |
| **17. Interview guide** | Semi-structured interview and FGD guides were used, adapted from existing tools. The manuscript does not mention pilot testing. |
| **18. Repeat interviews** | No repeat interviews were conducted. |
| **19. Audio/visual recording** | Yes, all interviews and FGDs were audio-recorded with participant consent. |
| **20. Field notes** | Yes, field notes were taken to capture non-verbal cues and served as a backup for audio recordings. |
| **21. Duration** | The manuscript does not specify exact durations, but sessions followed standard qualitative practice durations (typically 45–90 minutes). |
| **22. Data saturation** | Yes, FGDs continued until data saturation was reached. |
| **23. Transcripts returned** | No, transcripts were not returned to participants for comment or correction. |

**Domain 3: Analysis and findings**

| **Item** | **Response** |
| --- | --- |
| **24. Number of data coders** | The research team conducting thematic analysis and multiple coders were involved. |
| **25. Description of the coding tree** | Coding was guided by the Bowen et al. (2009) feasibility framework. |
| **26. Derivation of themes** | Themes were both deductively informed by the feasibility framework and inductively derived from participants' responses. |
| **27. Software** | NVivo software was used to manage and analyze the qualitative data. |
| **28. Participant checking** | No, participant checking (member validation) was not conducted. |
| **29. Quotations presented** | Yes, direct participant quotations were included and attributed by role and facility (e.g., "Postnatal woman, Katoro Health Centre"). |
| **30. Data and findings consistency** | Yes, the findings were well-supported by verbatim quotes that emerged from the data. |
| **31. Clarity of major themes** | Yes, major themes such as acceptability, feasibility, peer support, practicality, and integration—were clearly described. |
| **32. Clarity of minor themes** | Yes, minority views and nuanced concerns (e.g., discomfort in larger groups, space limitations, sustainability concerns) were presented and discussed. |
