# Supplementary material for: Perspectives of women, healthcare providers and health managers on Group Antenatal Care implementation in Geita, Tanzania: A qualitative study
Source: PLoS One. 2026 Apr 15;21(4):e0345539. doi: 10.1371/journal.pone.0345539 (PMC13082642; doi:10.1371/journal.pone.0345539)
Supplement: Attachment — Submitted filename: PONE-D-25-40423+Comments.pdf [file pone.0345539.s002.pdf]

PLOS ONE

Perspectives of women, health care providers and health managers on Group Antenatal Care (G-ANC) model implementation in Geita, Tanzania: A qualitative study

--Manuscript Draft--

**Manuscript Number:**	PONE-D-25-40423
**Article Type:**	Research Article
**Full Title:**	Perspectives of women, health care providers and health managers on Group Antenatal Care (G-ANC) model implementation in Geita, Tanzania: A qualitative study
**Short Title:**	Implementation of Group Antenatal Care (G-ANC) ~~model~~

**Corresponding Author:**	Alen Kinyina<br>Prime Health Initiative Tanzania (PHIT)<br>Dar es salaam, Tanzania TANZANIA, UNITED REPUBLIC OF

**Keywords:**	Antenatal care, Group antenatal, Pregnancy, ANC, Women

Abstract:

Background

Among the root cause of high maternal and neonatal mortality is low utilization of antenatal care services and sub-optimal quality of care during pregnancy. Group Antenatal Care (G-ANC) is an innovative model of care where pregnant women at similar stages of pregnancy meet for their care visits, rather than individually. This approach promotes peer support, shared learning, and a sense of community among pregnant women, while also enhancing the quality of care. This study aimed to assess the perspectives of providers, women and managers to determine the feasibility acceptability of G-ANC.

Methods

This qualitative study explored the perspectives of women, healthcare providers, and health managers on the acceptability and feasibility of G-ANC in Geita. Data were collected through six Focus Group Discussions (FGDs) with women who had recently attended G-ANC sessions, Key Informant Interviews (KIIs) with ANC providers and health managers. Transcripts were thematically analyzed using NVIVO software, guided by the Bowen et al.'s (2009) feasibility framework, focusing on perceptions, practicality, adaptability, integration, and acceptability.

Results

Findings revealed high levels of satisfaction and acceptability of G-ANC among all stakeholder groups. Women reported increased knowledge on maternal health, emotional support, and enhanced communication with providers. Healthcare providers noted improved efficiency, better patient engagement, and peer support among clients. Health managers observed increased ANC attendance and more interactive care. However, challenges such as limited space, need for facilitator training, and resource constraints were identified.

Conclusion

The G-ANC model is highly acceptable and feasible in Geita context. It demonstrated significant benefits for maternal health education, peer support, and healthcare delivery efficiency. These findings support the scale-up of G-ANC, contingent upon the allocation of resources towards infrastructure development, training of providers, and service integration. Institutionalizing G-ANC as a standard ANC model could be an option to address the low coverage of recommended visits.

**Order of Authors:**	Alen Kinyina
	Augustino Hellar
	Raymond Bandio
	Hamid Mandali
	Ahmad Makuwani
	Abubakari Munga
	Phineas Sospeter
	Yusuph Kulindwa

Powered by Editorial Manager® and ProduXion Manager® from Aries Systems Corporation

	Ntuli Kapologwe
	Frank Phiri
	Isaac Lyatuu
	Wilfred Kafuku
	Cypirian Mtani
	Husna Athuman
	James Hellar
	Omari Sukari
	James Tumain Kengia
	Erin Jones-Avni
	Hermes Gichane
**Additional Information:**	
**Question**	**Response**
**Financial Disclosure**<br><br>Enter a financial disclosure statement that describes the sources of funding for the work included in this submission. Review the submission guidelines for detailed requirements. View published research articles from *PLOS ONE* for specific examples.<br><br>This statement is required for submission and **will appear in the published article** if the submission is accepted. Please make sure it is accurate.<br><br>**Funded studies**<br>Enter a statement with the following details:<br>• Initials of the authors who received each award<br>• Grant numbers awarded to each author<br>• The full name of each funder<br>• URL of each funder website<br>• Did the sponsors or funders play any role in the study design, data collection and analysis, decision to publish, or preparation of the manuscript?<br><br>Did you receive funding for this work?	Yes
Please add funding details.<br>as follow-up to "**Financial Disclosure**	This project was funded by the Bill and Melinda Gates Foundation (INV-046249).

Powered by Editorial Manager® and ProduXion Manager® from Aries Systems Corporation

Enter a financial disclosure statement that describes the sources of funding for the work included in this submission. Review the submission guidelines for detailed requirements. View published research articles from *PLOS ONE* for specific examples.<br><br>This statement is required for submission and **will appear in the published article** if the submission is accepted. Please make sure it is accurate.<br><br>**Funded studies**<br>Enter a statement with the following details:<br>• Initials of the authors who received each award<br>• Grant numbers awarded to each author<br>• The full name of each funder<br>• URL of each funder website<br>• Did the sponsors or funders play any role in the study design, data collection and analysis, decision to publish, or preparation of the manuscript?<br><br>Did you receive funding for this work?"	
Please select the country of your main research funder (please select carefully as in some cases this is used in fee calculation).<br>as follow-up to "**Financial Disclosure**<br><br>Enter a financial disclosure statement that describes the sources of funding for the work included in this submission. Review the submission guidelines for detailed requirements. View published research articles from *PLOS ONE* for specific examples.<br><br>This statement is required for submission and **will appear in the published article** if the submission is accepted. Please make sure it is accurate.	UNITED STATES - US

**Funded studies**<br>Enter a statement with the following details:<br>• Initials of the authors who received each award<br>• Grant numbers awarded to each author<br>• The full name of each funder<br>• URL of each funder website<br>• Did the sponsors or funders play any role in the study design, data collection and analysis, decision to publish, or preparation of the manuscript?<br><br>Did you receive funding for this work?"	
**Competing Interests**<br><br>Use the instructions below to enter a competing interest statement for this submission. On behalf of all authors, disclose any competing interests that could be perceived to bias this work—acknowledging all financial support and any other relevant financial or non-financial competing interests.<br><br>This statement is required for submission and **will appear in the published article** if the submission is accepted. Please make sure it is accurate and that any funding sources listed in your Funding Information later in the submission form are also declared in your Financial Disclosure statement.<br><br>View published research articles from *PLOS ONE* for specific examples.	The authors have declared that no competing interests exist.

**NO authors have competing interests**<br><br>Enter: *The authors have declared that no competing interests exist.*<br><br>**Authors with competing interests**<br><br>Enter competing interest details beginning with this statement:<br><br>*I have read the journal's policy and the authors of this manuscript have the following competing interests: [insert competing interests here]*<br><br>* typeset	
**Ethics Statement**<br><br>Enter an ethics statement for this submission. This statement is required if the study involved:<br><br>• Human participants<br>• Human specimens or tissue<br>• Vertebrate animals or cephalopods<br>• Vertebrate embryos or tissues<br>• Field research<br><br>Write "N/A" if the submission does not require an ethics statement.<br><br>General guidance is provided below. Consult the submission guidelines for detailed instructions. **Make sure that all information entered here is included in the Methods section of the manuscript.**	Ethical considerations<br>The ethical approval for this project was sought from the National Institute of Medical Research (NIMR) Tanzania, under approval certificate number NIMR/HQ/R.8a/Vol.IX/4194 dated January 23rd, 2023. The team considered all necessary measures to ensure the recruitment, consenting, data collection, and publication of the materials related to this study are following regulations governing research standards. Key consenting processes were taken to ensure voluntary participation of the study participants, protection of their privacy, confidentiality of the data and their safety.<br>Informed consent<br>G-ANC was implemented within the framework of existing national antenatal care policies, utilizing healthcare providers from the participating health facilities. Oral consent was obtained from women prior to their engagement in G-ANC services, aligning with the standard procedures commonly followed during routine antenatal care.<br>For participants involved in the qualitative component of the study to explore their perceptions and experiences, both verbal and written informed consent were obtained prior to conducting the interviews. The consenting process was administered by trained research staff and conducted in Kiswahili (the national language) to ensure comprehension and cultural appropriateness. Each participant signed two copies of the consent form: one was retained by the participant for their records, and the second was securely stored by the study team. The consent forms included components outlining the purpose of the study, procedures involved, potential risks and benefits, confidentiality measures, voluntary participation, and the right to withdraw at any time without affecting their access to healthcare services. Participants were encouraged to ask questions, and their understanding was verified prior to signing the forms.

Powered by Editorial Manager® and ProduXion Manager® from Aries Systems Corporation

**Format for specific study types**<br><br>**Human Subject Research (involving human participants and/or tissue)**<br>• Give the name of the institutional review board or ethics committee that approved the study<br>• Include the approval number and/or a statement indicating approval of this research<br>• Indicate the form of consent obtained (written/oral) or the reason that consent was not obtained (e.g. the data were analyzed anonymously)<br><br>**Animal Research (involving vertebrate animals, embryos or tissues)**<br>• Provide the name of the Institutional Animal Care and Use Committee (IACUC) or other relevant ethics board that reviewed the study protocol, and indicate whether they approved this research or granted a formal waiver of ethical approval<br>• Include an approval number if one was obtained<br>• If the study involved *non-human primates*, add *additional details* about animal welfare and steps taken to ameliorate suffering<br>• If anesthesia, euthanasia, or any kind of animal sacrifice is part of the study, include briefly which substances and/or methods were applied<br><br>**Field Research**<br><br>Include the following details if this study involves the collection of plant, animal, or other materials from a natural setting:<br>• Field permit number<br>• Name of the institution or relevant body that granted permission	
**Data Availability**<br><br>Authors are required to make all data underlying the findings described fully available, without restriction, and from the time of publication. PLOS allows rare exceptions to address legal and ethical concerns. See the PLOS Data Policy and FAQ for detailed information.	No - some restrictions will apply

A Data Availability Statement describing where the data can be found is required at submission. Your answers to this question constitute the Data Availability Statement and **will be published in the article**, if accepted.<br><br>**Important:** Stating 'data available on request from the author' is not sufficient. If your data are only available upon request, select 'No' for the first question and explain your exceptional situation in the text box.<br><br>Do the authors confirm that all data underlying the findings described in their manuscript are fully available without restriction?	
**Describe where the data may be found in full sentences. If you are copying our sample text, replace any instances of XXX with the appropriate details.**<br><br>• If the data are **held or will be held in a public repository**, include URLs, accession numbers or DOIs. If this information will only be available after acceptance, indicate this by ticking the box below. For example: *All XXX files are available from the XXX database (accession number(s) XXX, XXX.).*<br>• If the data are all contained **within the manuscript and/or Supporting Information files**, enter the following: *All relevant data are within the manuscript and its Supporting Information files.*<br>• If neither of these applies but you are able to provide **details of access elsewhere**, with or without limitations, please do so. For example:<br><br>*Data cannot be shared publicly because of [XXX]. Data are available from the XXX Institutional Data Access / Ethics Committee (contact via XXX) for researchers who meet the criteria for access to confidential data.*<br><br>*The data underlying the results presented in the study are available from (include the name of the third party*	The data supporting the findings of this study are not publicly available due to institutional and national data protection policies. However, access to the data may be granted upon reasonable request and following the necessary organizational and administrative approvals. Interested researchers may contact the Prime Health Initiative Tanzania (PHIT) via the correspondent author of this manuscript at alen.kinyina@phit.or.tz for further information on the application process and conditions for data access. Access will be granted to qualified researchers for academic or policy-related purposes, subject to approval by the relevant ethics and administrative review bodies.

and contact information or URL).<br>• This text is appropriate if the data are owned by a third party and authors do not have permission to share the data.<br><br>* typeset	
Additional data availability information:	

Manuscript

Click here to access/download;Manuscript;Revised G-ANC Manuscript.docx

Perspectives of women, health care providers and health managers on Group Antenatal Care (G-ANC) model implementation in Geita, Tanzania: A qualitative study

Alen Kinyina[1,7], Augustino Hellar[1], Raymond Bandio[1], Hamid Mandali[1], Ahmad Makuwani[2], Abubakari Munga[1], Phineas Sospeter[2], Yusuph Kulindwa[1], Ntuli Kapologwe[4], Frank Phiri[1], Isaac Lyatuu[1], Wilfred Kafuku[1], Cyprian Mtani[1], Husna Athumani[1] James Hellar[2], Omari Sukari[3], James Tumani Kengia[5,6], Erin Jones-Avni[7], Hermes Gichane[7]

[1]Prime Health Initiative Tanzania (PHIT)
[2]Ministry of Health, Tanzania
[3]Geita Regional Health Management Team (RHMT)
[4]East, Central, and Southern Africa Health Community (ECSA-HC)
[5]President's Office Regional Administration and Local Government (PORALG)
[6]University of Dodoma, Tanzania (UDOM)
[7]London School of Hygiene and Tropical Medicine (LSHTM)

Abstract

Background

Among the root cause of high maternal and neonatal mortality is low utilization of antenatal care services and sub-optimal quality of care during pregnancy. Group Antenatal Care (G-ANC) is an innovative model of care where pregnant women at similar stages of pregnancy meet for their care visits, rather than individually. This approach promotes peer support, shared learning, and a sense of community among pregnant women, while also enhancing the quality of care. This study aimed to assess the perspectives of providers, women and managers to determine the feasibility acceptability of G-ANC.

Methods

This qualitative study explored the perspectives of women, healthcare providers, and health managers on the acceptability and feasibility of G-ANC in Geita. Data were collected through six Focus Group Discussions (FGDs) with women who had recently attended G-ANC sessions, Key Informant Interviews (KIIs) with ANC providers and health managers. Transcripts were thematically analyzed using NVIVO software, guided by the Bowen et al.'s (2009) feasibility framework, focusing on perceptions, practicality, adaptability, integration, and acceptability.

Results

Findings revealed high levels of satisfaction and acceptability of G-ANC among all stakeholder groups. Women reported increased knowledge on maternal health, emotional support, and enhanced

communication with providers. Healthcare providers noted improved efficiency, better patient engagement, and peer support among clients. Health managers observed increased ANC attendance and more interactive care. However, challenges such as limited space, need for facilitator training, and resource constraints were identified.

Conclusion

The G-ANC model is highly acceptable and feasible in Geita context. It demonstrated significant benefits for maternal health education, peer support, and healthcare delivery efficiency. These findings support the scale-up of G-ANC, contingent upon the allocation of resources towards infrastructure development, training of providers, and service integration. Institutionalizing G-ANC as a standard ANC model could be an option to address the low coverage of recommended visits.

Background

Maternal and neonatal mortality remain a pressing issue worldwide. Every two minutes, a woman dies from complications related to pregnancy or childbirth, and each day, 6,300 newborns die(1). Every year about 2.3 million newborns and 303,000 women die due to pregnancy or childbirth complications (1,2). According to the World Health Organisation (WHO), approximately more than 70% of maternal deaths occur in sub-Saharan Africa(3). In Tanzania, maternal deaths are about 104 per 100,000 live births which is higher than the SDGs target of 70 deaths per 100,000 live births(4). To improve the outcomes of pregnancy and newborn health, the WHO recommends adequate adherence to antenatal care (ANC) services (5,6).

Antenatal care refers to “care provided by skilled healthcare professionals to pregnant women and adolescent girls to ensure the best health conditions for both mother and baby during pregnancy(5). Adequate ANC services, including routine screening and diagnosis, reduce the frequency of maternal death, miscarriages, birth defects, low birth weight, neonatal infections, and other preventable health problems(7). Workload, lack of essential commodities, and demotivated health care providers lead to low satisfaction of services and poor utilisation of ANC services among pregnant women in countries with limited resources (6). In 2016, the WHO revised the ANC guideline, recommending each pregnant woman attend at least eight contacts instead of four visits(3). However, the coverage of recommended ANC visits and the provision of quality ANC services is still low in many developing countries. In Tanzania, the ANC coverage of at least four visits is less than 68% (4,5). As such, various approaches have been

taken to ensure high coverage of ANC visits, including the implementation of the innovative Group Antenatal Care (G-ANC) model (8,9)

G-ANC was originally implemented in high-resource settings and has been found to have a beneficial impact on pregnancy care, outcomes, and experience of care (10). This model provides health care to women in the group at the same time during group visits, as well as creating a support group of women at a similar stage in pregnancy. Groups of 8–15 women meet for group visits which include self-assessments, individual private clinical ANC consultations with the provider, and a facilitated, highly participatory group session to learn, share, and problem solve (7,11). With an enhanced experience of care and increasing health literacy, the quality of care and engagement of women in the ANC process encourage them to return for their scheduled visits (6,12). In addition, this has the potential to be more efficient, as the provider can provide information relevant to the particular stage of pregnancy to all women at the same time rather than to each woman individually (11–13). A key part of this model is that women do some of their own assessments (blood pressure, weight), with the provider as a facilitator, to encourage their investment in self-care and care of the foetus (6,12). Additionally, because the women are all at a similar stage of pregnancy, the GANC sessions develop a peer support network where women are receiving the same health information and sharing many of the same pregnancy experiences (11,12). Initial assessments in various countries have found this model to be highly desired by both women and providers, and it appears to lead to increased utilisation of health services and improved health literacy (6,7,10,11).

Because of increased interest in adaptation of G-ANC within low-resource settings, donors and policymakers have turned attention to this ANC service delivery model (10–12). In 2022, a pilot implementation project was introduced in Tanzania to assess the impact of G-ANC on prenatal service utilisation (14). Unpublished findings show that G-ANC enhanced the proportion of women completing recommended ANC visits from 31% in 2022 to 95% in 2024. In addition, women attending prenatal care through the G-ANC model demonstrated a high level of engagement and retention in care.

This highlights a significant opportunity and the potential of scaling up this intervention in order to increase ANC coverage, quality care, and ultimately reduce maternal and neonatal mortality. However, there has been no study conducted in Tanzania to assess the perspectives of women and providers regarding the implementation of G-ANC. The lack of studies on the acceptability of G-ANC in local contexts limits solid evidence informing feasibility of the scaling-up this intervention. Not understanding the perspectives of beneficiaries can pose limitations to influencing policymakers and stakeholders to adopt this intervention. Furthermore, scaling up an intervention that is not informed by evidence from

primary beneficiaries reduces the sense of ownership and engagement in the intervention which can lead to its failure. Therefore, this study was carried out to understand the perspectives of women, providers, and managers regarding the implementation of group antenatal care ~~in order~~ to gather the evidence to inform ANC policy ~~improvement for ending preventable maternal and neonatal deaths attributed to poor utilization of ANC services~~. If validated, ~~the~~ G-ANC ~~approach~~ can augment or ~~even~~ replace the current ~~traditional ANC approach~~.

Methodology

Qualitative study design

Given the exploratory nature of this research, the study design followed a qualitative approach. Qualitative study design is an appropriate method in assessing experience, views and perspectives about certain phenomena (15). Focused group discussions (FGDs) were conducted with women who attended G-ANC and service providers as key informants (KIIs) to explore their actual experience of G-ANC. The FGD and KII guides were used in gathering opinions and perspectives of the respondents on a variety of topics, including quality of ANC services during G-ANC. Modified interview guides were adopted from elsewhere (12). The project was implemented from 1 February 2023 to 1 June 2024. ~~However,~~ participant recruitment for the qualitative component commenced on 15 June 2024 and continued until 31 August 2024.

Selection of participants

Recently delivered women: ~~Six~~ facilities were purposefully selected (stratified by rural, peri-urban, -urban, and urban) for participation in FGD. Additionally, facilities were categorized as hospital-level, health center-level, and dispensary-level. One FGD was conducted with pregnant women or mothers who delivered within the past 6 months from each of the selected facilities' catchment areas until we reached saturation in the responses. The snowball sampling method was used to reach women who participated in G-ANC previously. Each FGD consisted of at most 8-12 women. KIIs were conducted with one ANC provider and the facility in-charge or health manager from all six facilities, for a maximum of 15 participants.

Data collection and analysis

FGDs and KIIs ~~interviews~~ were conducted in Swahili and all interviews were audio recorded. Translation from Swahili into English was done during transcription and kept secure. Semi-structured interviews and focus groups were conducted in private areas and by trained interviewers fluent in English and Kiswahili

who were not associated with the Ministry of Health. Data was collected through pre-developed interview guides, and flexibility was allowed in the formulation of questions and probing based on participant responses. In addition, field notes were taken to capture non-verbal observations and to act as backup in case of failure or malfunction of the recorder. Verbal consents were obtained to audio-record the sessions and those not willing to be recorded were ineligible to participate in the group discussions or interviews. Two data collectors were overseeing each interview: one as a moderator and another as a note-taker. The transcribed notes were imported from MS Word to NVIVO software, coded, and analyzed along the lines of the study objectives and questions. The analysis was conducted in relationship to perceived acceptability of interventions in terms of the Bowen et al. (2009) framework including perceptions, practicality, adaptability, integration, fit with values, and acceptability. Themes were identified using a combination of prior issues included in the discussion and interview guides that were informed by the research questions as well as emergent issues raised by the participants.

Ethical considerations

The ethical approval for this project was sought from the National Institute of Medical Research (NIMR) Tanzania, under approval certificate number NIMR/HQ/R.8a/Vol.IX/4194 dat[illegible]anuary 23rd, 2023. The team considered all necessary measures to ensure the recruitment, consenting, data collection, and publication of the materials related to this study are following regulations governing research standards. Key consenting processes were taken to ensure voluntary participation of the study participants, protection of their privacy, confidentiality of the data and their safety.

Informed [illegible]nsent

G-ANC was implemented within the framework of existing national antenatal care policies, utilizing healthcare providers from the participating health facilities. Oral consent was obtained from women prior to their engagement in G-ANC ~~services~~, aligning with the standard procedures commonly followed during routine antenatal care.

For participants involved in the qualitative component of the study to explore their perceptions and experiences, both verbal and written informed consent were obtained prior to conducting the interviews. The consenting process was administered by trained research staff and conducted in Kiswahili (the national language) to ensure comprehension and cultural appropriateness. Each participant signed two copies of the consent form: one was retained by the participant for their records, and the second was securely stored by the study team. The consent forms included components outlining the purpose of the study, procedures involved, potential risks and benefits, confidentiality measures, voluntary participation, and the right to withdraw at any time without affecting their access

to healthcare services. Participants were encouraged to ask questions, and their understanding was verified prior to signing the forms.

Results

Fifteen KIIs were conducted with ANC providers, facility in-charges from all six facilities, and health managers. A total of six FGDs were conducted, including four with postnatal women and two with pregnant women who were continuing with G-ANC. The views and perspectives of the various participants were organized into themes, as presented in this section.

Experience of women about Group Antenatal Care (G-ANC)

Women who participated in the qualitative study reported positive experiences, emphasizing the benefits of shared learning, emotional support, and improved access to healthcare services. G-ANC was recognized as an effective platform for empowering women with essential maternal health knowledge. Women expressed that attending G-ANC sessions helped them gain a deeper understanding of pregnancy-related issues, including nutrition, birth preparedness, and newborn care. One participant shared,

"Before joining the group sessions, I was not aware of the importance of balanced nutrition during pregnancy. Now, I not only eat better but also encourage other women in my community to do the same.". Postnatal woman, Katoro Health Centre

The interactive nature of G-ANC also created a sense of community among the women, reducing feelings of isolation during pregnancy. Some women appreciated the opportunity to share experiences and learn from one another.

"It felt reassuring to be in a group with other pregnant women who were going through the same journey as me," said one woman. *"We encouraged each other and became like a family."* Postnatal woman, Nzera DH

Women emphasized how G-ANC fosters a sense of belonging, emotional support, and collective learning, making pregnancy a more positive and informed experience for women. This support system played an important role in alleviating fears and anxieties related to pregnancy and childbirth as noted from some women who said:

> *"Listening to other women's experiences made me realize that I was not alone in my struggles. We shared advice, and it helped me feel more confident about my pregnancy."* Postnatal woman, Nkome Disp

"In the group, we laughed, learned, and supported each other. It was not just about medical check-ups; it was a place where we felt heard and understood." Postnatal woman, Katoro Health Center

"I used to feel anxious about childbirth, but hearing stories from other mothers who had gone through it helped ease my fears. Their experiences gave me strength and encouragement." Postnatal woman, Bwanga Health Center

Additionally, women noted that G-ANC improved their confidence in communicating with healthcare providers. This increased engagement contributed to better adherence to recommended antenatal care practices. They felt more comfortable asking questions and seeking clarification about their health. One participant explained,

"In the past, I was afraid to ask the nurse questions, but in the group, we openly discuss everything, and I feel more prepared for delivery." Postnatal woman, Chato DH

Perception and experience of healthcare providers on G-ANC

Healthcare providers who implemented and facilitated G-ANC reported their positive experiences, highlighting its effectiveness in improving maternal health outcomes, improving patient engagement, and fostering a supportive environment for both providers and pregnant women. HCWs acknowledged that G-ANC allowed them to deliver health education more efficiently, reaching multiple women at once instead of repeating the same information individually. One midwife shared,

"With G-ANC, we can educate many women at the same time, ensuring they receive consistent and accurate information. It saves time and improves understanding." HCP, Nzera DH

Another key benefit recognized by HCWs was the improved rapport and trust between providers and pregnant women. In traditional ANC, women often hesitate to ask questions due to time constraints or fear of being judged. Improved communication helped providers address misconceptions and ensure women were better prepared for childbirth. ~~However,~~ G-ANC created an open and interactive space where women felt comfortable discussing their concerns. A nurse explained,

"In one-on-one visits, many women remain silent, but in a group setting, they are more open to asking questions and sharing their concerns. It makes our work easier and more impactful." HCP, Nkome dispensary

HCWs also observed that G-ANC strengthened peer support among pregnant women and promoted positive behavioral changes. Women, inspired by their peers, became more proactive in adhering to recommended antenatal care practices, such as taking iron supplements, IPTp uptake, and preparing for institutional delivery. One health provider noted,

> *"Women who were initially reluctant to follow ANC recommendations became more motivated after seeing others share their positive experiences. The group setting created a sense of accountability and encouragement." Healthcare Provider,* Nzera DH

Despite these benefits, some HCWs mentioned challenges, such as the need for additional space and resources to facilitate group sessions. However, they believed the advantages far outweighed the challenges.

> *"The impact on maternal health is evident. Women are more informed, confident, and engaged. We need to invest more in scaling up this model," Facility In -charge,* Bwanga Health Centre

Health managers' perception and experience with G-ANC

Health managers (C/RHMTs) overseeing the implementation of services in Chato and Geita DC expressed strong support for the model, recognizing its transformative impact on maternal health care services. They highlighted improvements in service delivery, increased ANC attendance, and better engagement between healthcare providers and pregnant women. Some managers noted that G-ANC has led to more efficient use of resources and improved health outcomes. One district SRH coordinator explained,

> *"G-ANC helped our facilities to increase ANC attendance indicator. Women enjoy coming to the sessions because they receive care and learn from each other in a supportive environment."* CHMT Member, Chato DC

Health managers acknowledged that unlike the traditional ANC model, which often resulted in brief interactions between providers and clients, G-ANC enhanced a more interactive and trusting environment. One district nurse officer shared,

> *"~~At Nzera~~ hospital we have seen a shift in how women perceive ANC services. They are more engaged, ask more questions, and actively participate in discussions, which was not the case before...This change was attributed to the group-based approach, which encouraged collective learning and reduced fear or hesitation among pregnant women".* CHMT Member, Geita DC

Health managers also appreciated the efficiency of G-ANC in optimizing healthcare workers' time and reducing workload pressures. Instead of repeating the same health education topics individually, providers could deliver key messages to multiple women simultaneously. They also strongly supported scaling up G-ANC, recognizing its potential to improve maternal health outcomes.

A regional health officer noted,

> *"With limited staff, G-ANC helps us reach more women effectively. Providers can dedicate more time to meaningful discussions rather than rushing through individual consultations. The impact is clear…women are more informed, confident, and proactive about their health. We need to invest in infrastructure and training to sustain and expand this approach,"* CHMT Member, Geita DC

Feasibility and acceptability of Group Antenatal Care

Feasibility of G-ANC model was assessed based on the Bowen et al. (2009) (16) framework. This framework has several attributes of assessing feasibility of intervention, however, in this evaluation, four relevant themes emerged from the interview transcripts: perceptions/adaptions, practicality, integration and acceptability.

Practicality of Group Antenatal Care

G-ANC required careful planning to ensure that health facilities had the necessary resources to run group sessions effectively. Key resources included trained healthcare workers, sufficient space for group meetings, and the provision of essential materials such as educational tools (maternal booklets) and proper health records. Healthcare workers reported that organizing and facilitating G-ANC sessions required more initial time and effort compared to individual visits. One midwife noted,

> *"At first, it was challenging to organize the group sessions because we had to coordinate schedules in the G-ANC Cohort tracker or MTUHA and ensure that we had enough space. But now that we have the process in place, we are trying our best." Healthcare Provider,* Butengorumasa Disp

Training healthcare workers to facilitate G-ANC was important to its success. HCWs noted the importance of equipping them with the skills needed to manage group dynamics and address the diverse needs of pregnant women in a group setting. A nurse explained,

"The training we received in Katoro made a huge difference in how we conduct the sessions. It's not just about providing information, it's about managing the group and facilitating discussion." Healthcare Provider, Katoro HC

Integrating G-ANC into routine maternal health services was seen as a practical and beneficial approach to improve service delivery efficiency. It allowed healthcare workers to educate multiple women simultaneously while still providing individualized care and counseling when necessary. This was particularly important in areas with high patient volumes, where traditional one-on-one consultations could lead to long waiting times and overcrowding. One facility in-charge said,

"G-ANC allows us to manage our time better. It reduces waiting times for women and ensures that we can reach more women with the same resources, especially in facilities with many clients ~~like Bwanga and Katoro~~ I think..." Facility In-charge, Nkome Disp

Integration with other maternal health services in G-ANC

The integration of G-ANC with other maternal, child health, and community services was a key feature of its implementation. Through leveraging existing health systems and services, G-ANC promoted a holistic approach to care. The integration was achieved through strategic collaboration between healthcare workers from different units such as labour ward and the laboratory. Another area of integration is family planning services. During G-ANC sessions, healthcare workers introduced a full session discussion about postpartum family planning, which allowed women to make informed decisions about their reproductive health before delivery. A healthcare worker explained,

"We ensure that women understand their options for family planning after childbirth.We encourage them to choose the methods before birth and this is a full session discussion". Healthcare Provider, Chato DH

Women, in turn, appreciated the opportunity to receive family planning counselling during their antenatal care period. A participant shared,

"It's helpful to learn about family planning while I am still pregnant. I feel more prepared for after delivery." Pregnant woman, Butengorumasa Disp

G-ANC's integration with community health services ensures continuity of care, especially in rural and hard-to-reach areas. Community health workers were actively involved in mobilizing pregnant women for group sessions, providing post-session follow-ups, and offering health education in the community. A CHW shared,

"I visit the women after they attend their G-ANC sessions to remind them about the next steps and encourage them to keep up with their health routines. Also, in Chato we do outreach sometimes too and we have some G-ANC sessions." CHW, Chato DH

Acceptability of Group Antenatal Care

For women, G-ANC was seen as a valuable alternative to traditional individual antenatal care. Many women appreciated the opportunity to engage with other pregnant women, which fostered a sense of community and emotional support. A woman attending G-ANC sessions shared,

> *"I feel less alone now because we talk about our pregnancies together, and I can learn from others' experiences. It makes me more confident about my pregnancy."* Postnatal woman, Nkome dispensary.

The shared learning environment allowed women to feel more empowered in managing their pregnancies, and the group format was often described as both informative and reassuring. One woman commented,

> *"I used to feel anxious going to individual appointments, but in a group, we share our worries and talk openly. It feels like we're all in it together."* Postnatal woman, Bwanga Health Center

While most women were positive about G-ANC, there were some who expressed concerns about group dynamics. One participant mentioned feeling shy or uncomfortable discussing personal health issues in front of others, especially in larger groups. However, the majority of women acknowledged that the benefits of being in a group far outweighed these concerns. This participant noted,

> *"Sometimes, I feel uncomfortable asking certain questions in front of so many people. It would be better if we had smaller groups."* Postnatal woman, Katoro Health Center

Acceptance of healthcare workers

Generally, healthcare workers who were involved in this evaluation accepted G-ANC as an effective method for delivering antenatal care. They recognized the advantages of the group model in terms of time management and increased efficiency. Also, they appreciated the opportunity to provide more interactive, participatory sessions. A midwife expressed,

> *"G-ANC has allowed us to engage with many women at once, which helps reduce the pressure on our time. It's a more organized way to deliver care, women feel more involved in their care,*

and it's much easier to address common questions in a group than one by one." Healthcare Provider, Nzera HC

However, some healthcare workers mentioned the initial learning challenges associated with facilitating group sessions. One healthcare worker explained,

> *"At first, we were unsure about how to manage the group dynamics and ensure every woman got the individual attention they needed. With more experience, we've found ways to balance the group discussions with one-on-one support."* Healthcare Provider, Nzera DH

Acceptance of G-ANC by the health managers

District Health Coordinators were generally supportive of G-ANC, as they saw the model as a means to increase service delivery coverage and improve pregnant outcomes. A CHMT member noted,

> *"G-ANC has increased the number of women attending antenatal care regularly which is a big win for our Geita region. It also reduces the workload on individual healthcare appointments."* CHMT member, Chato DC

It was recognized that the model could potentially reduce maternal and neonatal health risks by increasing the number of women attending regular check-ups and receiving timely care. However, some DHCs expressed concerns about the initial costs of implementing G-ANC, particularly after the Mlinde Mama project closeout. A DHC mentioned,

> *"While we support the idea, the costs involved in setting up the program is a bit challenging especially if PHIT leave the project. But we saw the value it brought in terms of both health outcomes and service efficiency, but we don't have money to print maternal booklets and to replace rechargeable batteries for BP machines."* CHMT member, Geita DC

Discussion

This study explored the perspectives of recently delivered women, healthcare providers, and health system managers about G-ANC in Geita. The present study demonstrate that G-ANC is a highly acceptable model that improves women's engagement, knowledge, emotional well-being during pregnancy, and enhances provider-client interaction in healthcare delivery. The findings are consistent with previous studies in sub-Saharan Africa and other low-resource settings that have demonstrated the

benefits of G-ANC in improving maternal health outcomes, health literacy, and service satisfaction (6,11,12,17). The shared positive experiences reported by women in this study align with findings from Ghana and Kenya, where G-ANC fostered social cohesion, peer support, and improved health literacy (6,12). This may be attributed by interactive learning environment and emotional support fostered through shared experiences.

Moreover, the enhancement of communication and trust between pregnant women and providers observed in this study corroborates evidence from a study in Nigeria and Kenya, where G-ANC increased client-provider interaction and improved understanding of maternal issues (11). This reinforces the notion that G-ANC, through its participatory design, facilitates more meaningful engagement compared to traditional one-on-one care. However, our study also highlights a unique value in which women reported gaining confidence in communicating with providers advocating for maternal health within their communities. This community-based ripple effect has not been extensively documented in previous studies and warrants the need of further exploration. Additionally, health managers and providers in this study recognized G-ANC as a model for increasing ANC attendance, improving quality of care, and reducing workload. These findings are consistent with other studies emphasizing the importance of service delivery models that optimize health worker time in low-resource settings. The findings demonstrate that G-ANC is feasible and practical with appropriate planning, trained HCPs, and adequate space for meetings (5,6,10,13,17).

The integration of G-ANC with postpartum family planning, CHWs support systems, and other maternal health services, as noted in our findings, illustrates the model's potential to offer holistic support. This aligns with Zielinski et al. (6), who advocated for integrated antenatal care systems that respond to the holistic needs of pregnant women. While the majority of participants endorsed G-ANC, some women expressed concerns about discussing personal health issues in group settings. This hesitation reflects cultural norms regarding privacy and is consistent with findings from a study in Kenya, where women preferred individual consultations for sensitive topics (12). However, unlike in some other settings where such discomfort significantly impacted participation, our study found that most women overcame initial shyness and eventually valued the shared experience. Interestingly, this study did not highlight significant male involvement in ANC through G-ANC. This is contrary to the previous evidence from Malawi showing that group care encouraged male participation in SRH services (8). The absence of this theme could suggest contextual or cultural differences in partner involvement in maternal health in Geita or a limitation in how group sessions were structured.

The findings of this study demonstrated acceptability of G-ANC among women and providers which highlights its potential for scale-up, particularly in regions where ANC coverage and maternal health

indicators remain suboptimal. The model appears to foster better maternal engagement, improve quality of care, and enhance satisfaction of services. Second, integration of other health messages with G-ANC strengthens the maternal health continuum and presents an opportunity to address multiple SRHR issues in a unified platform. This aligns with global strategies for comprehensive, people-centered health systems. The study adds to the growing evidence that G-ANC is not merely a service delivery innovation but a transformative approach that fosters peer-to-peer learning and empowerment of women for self-care.

While the study generated valuable insights, it has several limitations. First, the data is drawn from a specific geographical context (six facilities), which may limit generalizability to other regions with different sociocultural dynamics or health system capacities. Future studies could expand to include a more diverse sample from multiple facilities. However, while the study utilized the Bowen et al. (16) framework for feasibility, the analysis did not explore economic feasibility or cost-effectiveness, which are crucial for informing national scale-up. A mixed-methods approach incorporating quantitative outcomes (e.g. cost-benefit analysis) would provide a more comprehensive picture.

Conclusion

Findings revealed high levels of satisfaction and acceptability of G-ANC among all stakeholder groups. Women reported increased knowledge on maternal health, emotional support, and enhanced communication with providers. Healthcare providers noted improved efficiency, better patient engagement, and peer support among clients. Health managers observed increased ANC attendance and more interactive care. However, challenges such as limited space, need for facilitator training, and resource constraints were identified. The G-ANC model is highly feasible for implementation in the Tanzanian context, with significant benefits for peer support and service delivery efficiency. These findings support the scale-up of G-ANC, provided investments are made in infrastructure, training of providers, and service integration. Institutionalizing G-ANC as a standard antenatal care model could be the best option to address the low coverage of ANC visits, suboptimal quality care, institutional deliveries.

KEY MESSAGES

What is already known on this topic:

- G-ANC) has been implemented in various settings globally, showing improvements in maternal health literacy, peer support, and satisfaction with care.
- There is limited research from other low-resource settings. However, available evidence suggests that G-ANC can improve antenatal care attendance and enhance provider-client interaction.
- Despite growing global interest, limited evidence exists on the acceptability and feasibility of G-ANC in routine health systems in Tanzania.

What this study adds:

- This study provides context-specific insights from women, healthcare providers, and health managers in Tanzania, showing that G-ANC is both feasible and highly acceptable.
- The study highlights how G-ANC improves emotional support, communication with providers, and women's engagement in care.
- It also identifies operational enablers and barriers including space constraints, need for training, and integration with other services—critical for successful scale-up.

How this study might affect research, practice, or policy

- The findings offer strong evidence to support the institutionalization of G-ANC as part of routine antenatal services in Tanzania.
- Policymakers and implementers can use these findings to inform national scale-up plans, resource allocation, and integration with broader maternal and newborn health programs.
- The study underscores the need for investment in provider training, infrastructure, and supportive supervision to sustain the quality and impact of G-ANC.

Funding

This project was funded by the Bill and Melinda Gates Foundation (BMGF)- INV-046249

Data Availability Statement

The data supporting the findings of this study are not publicly available due to institutional and national data protection policies. However, access to the data may be granted upon reasonable request and following the necessary organizational and administrative approvals. Interested researchers may contact the Prime Health Initiative Tanzania (PHIT) via the correspondent author of this manuscript at alen.kinyina@phit.or.tz for further information on the application process and conditions for data access. Access will be granted to qualified researchers for academic or policy-related purposes, subject to approval by the relevant ethics and administrative review bodies.

Acknowledgments

We extend our heartfelt appreciation to the healthcare providers, facility in-charges, and health managers at the participating health facilities in Geita Region for their invaluable time, cooperation, and commitment to this research. Special thanks go to the Geita Regional Health Management Team (RHMT) and the Council Health Management Teams (CHMTs) of Chato and Geita DC for their technical and logistical support throughout the study period.

We acknowledge the support of the Ministry of Health (MoH) and the President Office - Regional Administration and Local Government (PORALG) for their policy guidance and facilitation of approvals essential for data collection and implementation of the G-ANC model in the public facilities.

Competing interests

All authors declare that they have no competing interests.

References

1. Maugeri A, Barchitta M, Schillaci G, Agodi A. Spatial patterns and temporal trends in stillbirth, neonatal, and infant mortality: an exploration of country-level data from 2000 to 2021. J Glob Health. 2025 Feb 21;15:04034.

2. Taher TMJ. Maternal Mortality: What is The Situation and The Determinants? Maaen Journal for Medical Sciences. 2024 Feb 3;3(1).

3. Fagbamigbe AF, Olaseinde O, Setlhare V. Sub-national analysis and determinants of numbers of antenatal care contacts in Nigeria: assessing the compliance with the WHO recommended standard guidelines. BMC Pregnancy Childbirth. 2021 Dec 25;21(1):402.

4. Tanzania Demographic and Health Survey and Malaria Indicator Survey (TDHS-MIS). 2022.

5. Camacho EM, Smyth R, Danna VA, Kimaro D, Kuzenza F, Laisser R, et al. Women's preferences for antenatal care in Tanzania: a discrete choice experiment. BMC Pregnancy Childbirth. 2022 Dec 7;22(1):296.

6. Zielinski R, Kukula V, Apetorgbor V, Awini E, Moyer C, Badu-Gyan G, et al. "With group antenatal care, pregnant women know they are not alone": The process evaluation of a group antenatal care intervention in Ghana. PLoS One. 2023 Nov 7;18(11):e0291855.

7. Grenier L, Suhowatsky S, Kabue MM, Noguchi LM, Mohan D, Karnad SR, et al. Impact of group antenatal care (G-ANC) versus individual antenatal care (ANC) on quality of care, ANC attendance and facility-based delivery: A pragmatic cluster-randomized controlled trial in Kenya and Nigeria. PLoS One. 2019 Oct 2;14(10):e0222177.

8. Jeremiah RD, Patel DR, Chirwa E, Kapito E, Mei X, McCreary LL, et al. A randomized group antenatal care pilot showed increased partner communication and partner HIV testing during pregnancy in Malawi and Tanzania. BMC Pregnancy Childbirth. 2021 Dec 24;21(1):790.

9. Munsey A, Kinyina A, Assenga M, Almeida A, Kitojo C, Reaves E, et al. Assessing the utility of pregnant women as a sentinel surveillance population for malaria in Geita, Tanzania, 2019-2021. International Journal of Infectious Diseases. 2023;136:57–63.

10. Liu Y, Wang Y, Wu Y, Chen X, Bai J. Effectiveness of the CenteringPregnancy program on maternal and birth outcomes: A systematic review and meta-analysis. Int J Nurs Stud. 2021 Aug;120:103981.

11. Noguchi L, Grenier L, Kabue M, Ugwa E, Oyetunji J, Suhowatsky S, et al. Effect of group versus individual antenatal care on uptake of intermittent prophylactic treatment of malaria in pregnancy and related malaria outcomes in Nigeria and Kenya: analysis of data from a pragmatic cluster randomized trial. Malar J. 2020 Dec 29;19(1):51.

12. Grenier L, Onguti B, Whiting-Collins LJ, Omanga E, Suhowatsky S, Winch PJ. Transforming women's and providers' experience of care for improved outcomes: A theory of change for group antenatal care in Kenya and Nigeria. PLoS One. 2022 May 3;17(5):e0265174.

13. Sayinzoga F, Lundeen T, Musange SF, Butrick E, Nzeyimana D, Murindahabi N, et al. Assessing the impact of group antenatal care on gestational length in Rwanda: A cluster-randomized trial. PLoS One. 2021 Feb 2;16(2):e0246442.

14. Hellar A, Kinyina A, Sospeter P, Kulindwa Y, Phiri F, Bandio R, et al. An assessment to inform programming for antenatal care services in six health facilities in Geita Region, Tanzania: a cross-sectional baseline survey. 2024.

15. Busetto L, Wick W, Gumbinger C. How to use and assess qualitative research methods. Neurol Res Pract. 2020 Dec 27;2(1):14.

16. Bowen DJ, Kreuter M, Spring B, Cofta-Woerpel L, Linnan L, Weiner D, et al. How we design feasibility studies. Am J Prev Med. 2009;36(5):452–7.

17. Lori JR, Ofosu-Darkwah H, Boyd CJ, Banerjee T, Adanu RMK. Improving health literacy through group antenatal care: a prospective cohort study. BMC Pregnancy Childbirth. 2017 Dec 14;17(1):228.

COREQ Checklist

Click here to access/download
Supporting Information
COREQ checklist.docx

Click here to access/download
Preprint PDF
Priprint Gates Foundation.pdf
